# The angiotensin II receptors type 1 and 2 modulate astrocytes and their crosstalk with microglia and neurons in an in vitro model of ischemic stroke

**DOI:** 10.1186/s12868-024-00876-x

**Published:** 2024-06-26

**Authors:** Daniel Navin Olschewski, Nilufar Nazarzadeh, Felix Lange, Anna Maria Koenig, Christina Kulka, Jella-Andrea Abraham, Stefan Johannes Blaschke, Rudolf Merkel, Bernd Hoffmann, Gereon Rudolf Fink, Michael Schroeter, Maria Adele Rueger, Sabine Ulrike Vay

**Affiliations:** 1 Department of Neurology, Faculty of Medicine and University Hospital of Cologne, University of Cologne, Cologne, Germany; 2 Cognitive Neuroscience, Institute of Neuroscience and Medicine (INM-3), Research Centre Juelich, Juelich, Germany; 3 Department of Mechanobiology, Institute of Biological Information Processing (IBI-2), Research Centre Juelich, Juelich, Germany

**Keywords:** Neuroinflammation, Astrocytes, Microglia, Cortical neuronal network, Cerebral ischemia, Renin–angiotensin–aldosterone system, Microelectrode array, MEA, Telmisartan, PD123319

## Abstract

**Background:**

Astrocytes are the most abundant cell type of the central nervous system and are fundamentally involved in homeostasis, neuroprotection, and synaptic plasticity. This regulatory function of astrocytes on their neighboring cells in the healthy brain is subject of current research. In the ischemic brain we assume disease specific differences in astrocytic acting. The renin–angiotensin–aldosterone system regulates arterial blood pressure through endothelial cells and perivascular musculature. Moreover, astrocytes express angiotensin II type 1 and 2 receptors. However, their role in astrocytic function has not yet been fully elucidated. We hypothesized that the angiotensin II receptors impact astrocyte function as revealed in an in vitro system mimicking cerebral ischemia.

Astrocytes derived from neonatal wistar rats were exposed to telmisartan (angiotensin II type 1 receptor-blocker) or PD123319 (angiotensin II type 2 receptor-blocker) under normal conditions (control) or deprivation from oxygen and glucose. Conditioned medium (CM) of astrocytes was harvested to elucidate astrocyte-mediated indirect effects on microglia and cortical neurons.

**Result:**

The blockade of angiotensin II type 1 receptor by telmisartan increased the survival of astrocytes during ischemic conditions in vitro without affecting their proliferation rate or disturbing their expression of S100A10, a marker of activation. The inhibition of the angiotensin II type 2 receptor pathway by PD123319 resulted in both increased expression of S100A10 and proliferation rate. The CM of telmisartan-treated astrocytes reduced the expression of pro-inflammatory mediators with simultaneous increase of anti-inflammatory markers in microglia. Increased neuronal activity was observed after treatment of neurons with CM of telmisartan- as well as PD123319-stimulated astrocytes.

**Conclusion:**

Data show that angiotensin II receptors have functional relevance for astrocytes that differs in healthy and ischemic conditions and effects surrounding microglia and neuronal activity via secretory signals. Above that, this work emphasizes the strong interference of the different cells in the CNS and that targeting astrocytes might serve as a therapeutic strategy to influence the acting of glia-neuronal network in de- and regenerative context.

**Supplementary Information:**

The online version contains supplementary material available at 10.1186/s12868-024-00876-x.

## Introduction

Astrocytes are the most abundant cell type of the central nervous system (CNS) and are involved in brain homeostasis, neuroprotection, regulation of synaptic plasticity, and blood–brain barrier maintenance. They interact with other cell types through various secretory mediators, ultimately playing an essential role in controlling the innate immune responses in the CNS [1–3]. During neuroinflammation, however, astrocytes display significant changes in phenotype, functions, and gene expression [4, 5]. Recent in vitro experiments of ischemic stroke have suggested a feedforward mechanism of activation of astrocytes and microglia, initiating avalanche-like neuroinflammatory responses and, consequently, neuronal damage [6]. Additionally, transcriptomic studies have revealed that astrocytes can adopt different phenotypes [7, 8]. However, a detailed categorization of astrocytes matching the gene expression profile with functional analyses is desired [9, 10].

The renin–angiotensin–aldosterone system (RAAS) plays a prominent role in regulating fluid and electrolyte balance, arterial pressure, and the autonomic system in the central nervous system (CNS) [11–13]. Its main effector is the octapeptide angiotensin II, which interacts mainly through two G protein-coupled receptors: the angiotensin II receptor, type 1 (AT1), and the angiotensin II receptor, type 2 (AT2). AT1 and AT2 are the most expressed angiotensin receptors, have low sequence identity and exhibit distinct pharmacological profiles. Angiotensin II predominantly acts through the activation of AT1 by inducing vasoconstriction and cellular growth while AT2 counteracts the action of AT1 through antiproliferative and vasodilative effects [14]. The effect mediated by AT1 can be inhibited through specific and competitive angiotensin II receptor blockers called sartans that have also been described to exert beneficial effects on inflammatory and metabolic processes [15]. Additionally, further angiotensin receptors have been identified (AT4, MasR, MrgD and ACE2) in the last two decades [16, 17]. As all components of the RAAS have been suggested to exist in individual tissue, it is possible to observe local production, release, and action of angiotensin II in brain cells such as glia and neural cells [18, 19]. This suggests a crucial role for these cells in the central effects of angiotensin II [19]. Mounting evidence shows that the RAAS is involved in the pathogenesis of numerous diseases of the CNS by increasing neuroinflammation [11]. For instance, AT1 stimulation has been shown to be associated with brain ischemia, blood–brain barrier breakdown, and inflammation [11, 16, 20].

Astrocytes have been identified as the major source of angiotensinogen within the brain [21, 22]. AT1 and AT2 are the predominantly expressed receptors on astrocytes and are expressed on the cell surface. Additionally, the expression of these two receptors and MasR has been described on mitochondria and cell nucleus of astrocytes [16, 23]. For instance, AT1 has been shown to be involved in the development of long-lasting, region-dependent and oxidative stress-independent astrocyte morphological alterations induced by Ketamine [24]. Furthermore, human astrocytes have shown to interact with their surrounding cells such as microglia by releasing the soluble form of aminopeptidase N as a component of the brain RAAS during neuroinflammation, enhancing microglia activation [1]. Additionally, a study showed that the decrease in dopamine levels observed in early stages of Parkinson’s disease and aging may promote neuroinflammation through increased AT1 expression with simultaneous decrease in AT2 expression while also affecting the regulation of RAAS activity in microglia by upregulation of the astrocyte-derived angiotensin II as well as via dopamine-induced regulation of microglial angiotensin receptors [25]. Focusing on the interaction between astrocytes and neurons, AT1 inhibition showed reduction in astrocyte activation and blood-brain barrier damage in epilepsy while attenuating consecutively seizure spike activity [26]. Furthermore, it has been suggested that astrocytic calcium response is increased through AT1 activation, altering cerebral blood flow in response to neuronal activity [27].

The angiotensin II receptors have therefore been suggested as potential therapeutic targets for several diseases, including cerebral ischemia [28].

In this study, we investigate the modulating mechanisms of angiotensin receptor blockers (ARBs) on astrocytes and their functional implications for neurons and microglia, exploring avenues for potential future therapies to enhance regenerative capacity in stroke patients.

Based on previous observations, we hypothesized that the modulation of angiotensin receptors on astrocytes significantly impacts the latter’s phenotype, survival, proliferation, and secretion of soluble factors that, in turn, interact with microglia and neurons. Therefore, we examined the effects of ARBs on resting primary astrocytes derived from neonatal rats and on astrocytes undergoing in vitro stroke by oxygen–glucose deprivation (OGD). To comprehend the functional implications for neurons and microglia, we further investigated the astrocytes’ consecutive effect on the expression of microglial phenotypes and neuronal functionality.

## Materials and Methods

All animal procedures were carried out according to the German Laws for Animal Protection. The local animal care committee (Tierschutz-Beauftragte University of Cologne) and governmental authorities (AZ UniKöln_Anzeige §4.16.021) had approved the study. Wistar rats for breeding were bought from Janvier Laboratories (Le Genest-Saint-Isle, France) and maintained in polysulfone cages with sawdust bedding on a 12 h light/dark cycle. Food and water were provided ad libitum in pathogen-free conditions. Eight different litters obtained from two breeding pairs were used for the experiments, and all pups of each litter (9–15) were pooled for cultures independent of their sex. Wistar rats for tissue harvesting of primary cortical neurons were obtained from Charles River Laboratories (Sulzfeld, Germany) and processed by the Institute of Biological Information Processing (IBI-2), Department of Mechanobiology, Research Center Juelich (Animal testing license: 84-02.04.2015.A173, LANUV NRW, Germany).

### Glia cell culture

Pure neonatal astrocyte and microglia cultures were harvested from the cortices of neonatal Wistar rats (P0–P3), which were sacrificed by unanesthetized decapitation [3, 29]. Briefly, cortices were dissected and blood vessels, meninges and white matter removed. Consecutively, the cortical tissue was cut into small pieces and incubated in 0.05% trypsin/0.02% EDTA solution (Thermo Fisher Scientific, Waltham, USA) for 15 min at 37 °C. The reaction was neutralized by the addition of cell culture media (Dulbecco’s essential media (DMEM) containing 10% fetal calf serum (FCS), 1% penicillin/streptomycin, and 2 mM l-glutamine) (Thermo Fisher Scientific, Waltham, USA). The cortices were dissociated by up- and down-pipetting. The resulting cell suspension was centrifuged at 1200 rpm for 2 min, and the cell pellets were resuspended in culture media and grown at 37 °C with 5% CO_2_. The culture medium was changed on the third day. Through a prolonged cultivation method, selective growth of astrocytes and microglia was ensured. After 8–10 days, cultures were shaken for 1 h at 250 rpm on an orbital shaker (37 °C) to detach microglia. The medium containing the layer of detached microglia was collected and immediately centrifuged at 1200 rpm for 2 min. After removing the supernatant, the obtained microglia pellet was resuspended in fresh culture medium and seeded into subcultures for further experiments (see below). Flasks were refilled with culture medium, and microglia harvesting was repeated three times at intervals of 3 days.

The remaining astrocytes in the flasks were detached using 0.05% trypsin/0.02% EDTA solution for 15 min at 37 °C and centrifuged for 3 min at 1200 rpm. The obtained astrocyte pellet was resuspended in fresh culture medium and sown into subcultures. All experiments were performed with these purified astrocyte cultures. All experiments were conducted in at least three biological triplicates that—in turn—were conducted with cell cultures out of at least three different litters.

### Treatment of primary astrocytes

Three to five days after seeding, astrocytes were incubated with either telmisartan at 0.1 µM, 1 µM, 10 µM, respectively, (CAS# 144701-48-4, Cayman Chemical Company, Michigan, USA) or PD123319 at 10 µM (CAS# 136676-91-0, Sigma Aldrich, Taufkirchen, Germany) for 48 h. Consecutively, RNA was isolated, live dead or ATP-assays were performed, glutamate concentration was measured in the cell culture medium, or cells were later exposed to a deprivation of oxygen and glucose (see below). The telmisartan concentrations corresponded to the range of drug concentrations previously found in the human plasma after intravenous administration and in the brain and plasma of mice after oral administration of telmisartan at 2 g/kg chow diet [30, 31]. Furthermore, we chose telmisartan, as it has the most prolonged half-life of all sartans with a mean of 24 h and the strongest binding affinity to AT1 [32]. The concentration of PD123319 corresponded to previous experiments [33–35]. PD123319 was dissolved in ddH_2_O. As telmisartan was dissolved in 0.5% dimethyl sulfoxide (DMSO; CAS# 67-68-5, Merck, Darmstadt, Germany), the other samples (control and PD123319-stimulated astrocytes) additionally received DMSO at a final concentration of 0.5% during experiments.

To obtain the supernatant of untreated control or stimulated astrocytes for the experiments with neurons and microglia, astrocytes were treated for 48 h with telmisartan or PD123319. Consecutively, medium was replaced with fresh culture medium without angiotensin 2 receptor blockers. After an additional 24 h, this so-called conditioned medium (CM) of the astrocytes was collected and used for further experiments.

### Oxygen–glucose deprivation

To simulate an in-vitro stroke model, we exposed astrocytes to a combined oxygen–glucose deprivation (OGD) as previously described [29]. Astrocytes were treated with telmisartan or PD123319 for 48 h, and, in order to induce OGD, culture medium was removed, and cells were washed with 1 × PBS. Then, cells were placed in a hypoxic chamber (Electrotek, Shipley, UK), and deoxygenated DMEM without glucose (Thermo Fisher Scientific, Waltham, USA) was added with insufflation of gas containing 80% N_2_, 10% CO_2_, and 10% H_2_ at 37 °C (Linde plc, Dublin, Republic of Ireland). During this time, neither telmisartan nor PD123319 was present in the culture. The oxygen concentration was determined with an oxygen meter (GMH 3611-GL, Greisinger, Regenstauf, Germany), ensuring an oxygen concentration below 0.1% for all experiments. After 2 h or 8 h, depending on the duration of the respective experiment, cells were removed from the chamber and left to recover in fresh glucose- and oxygen-containing culture medium under normoxia (5% CO_2_) without telmisartan or PD123319 at 37 °C. After 24 h, those recovered cultures were used for further experiments. For control conditions, the cells were merely washed with 1 × PBS before receiving fresh culture medium. For the following experiments, we term the control cells “untreated astrocytes” and the OGD-exposed cells that underwent OGD as “OGD-treated astrocytes”.

### Microglia treatment

Following 24 h after seeding in subcultures, microglia were exposed to CM from untreated astrocytes (CM Control), CM from astrocytes stimulated with 10 µM PD123319 (CM PD123319) or CM from astrocytes stimulated with 10 µM telmisartan (CM Telmisartan) over 24 h. Consecutively, RNA was isolated or microglia were fixed with 4% paraformaldehyde (PFA) for immunocytochemical staining.

### Human embryonic kidney cells 293 (HEK293)

HEK293 cells were cultured from frozen stocks at 37 °C in DMEM (Thermo Fisher Scientific, Waltham, USA) supplemented with 10% fetal calf serum (FCS), 1% penicillin/streptomycin, and 2 mM l-glutamine [36]. At a confluency of 80%, cells were regularly split and seeded for experiments. HEK293 cells were used for immunocytochemistry and RNA isolation and used as a negative control for angiotensin receptor expression.

### Primary cortical neurons

Embryonic rat cortical neurons were obtained from pregnant rats (Wistar, Charles River, Sulzfeld, Germany) at 18–19 days of gestation [37]. Briefly, after anesthetizing the pregnant rat with CO_2_ and decapitating it, the embryos were surgically removed from the adult rat and kept in ice-cold Hanks’ Balanced Salt Solution (Sigma Aldrich, Taufkirchen, Germany). Cortices were isolated from embryonic brains and further trypsinized using 0.05% trypsin/0.02% EDTA solution (Thermo Fisher Scientific, Waltham, USA) for 15 min at 37 °C. The tissue was transferred to prewarmed neurobasal media (Thermo Fisher Scientific, Waltham, USA), supplemented with 1% GlutaMAX (Thermo Fisher Scientific, Waltham, USA), 2% B-27 (Thermo Fisher Scientific, Waltham, USA), and 0.1% Gentamicin (Sigma, Taufkirchen, Germany). Cortices were washed three times to remove any remaining trypsin solution. Consecutively, cortical cells were dissociated by trituration. A number of 80 cells/mm^2^ was seeded in 1 ml of neurobasal media and on microelectrode arrays (60MEA200/30iR-T, Multi Channel Systems (MCS), Reutlingen, Germany), which were pre-coated with 15% poly-l-ornithine diluted with ultrapure water (Sigma, Taufkirchen, Germany) and 1.25% fibronectin diluted with 1 × PBS (R&D Systems, Minneapolis, USA). Half of the medium was changed every 2 days. All experiments were conducted in at least three biological triplicates that—in turn—were conducted with cell cultures out of at least three different litters.

### Microelectrode arrays (MEAs)

Cortical neurons were cultured for 15–30 days until mature bursting and synchronic activity were achieved in at least 10 electrodes within the network. For the experiments, half of the neurobasal media was removed and replaced with CM of unstimulated astrocytes (CM Co), 10 µM telmisartan- (CM Telmisartan) or 10 µM PD123319-(CM PD123319) stimulated astrocytes. A naïve control group (Ctrl) received the same volume of neurobasal media. Measurements were performed shortly before the addition of CM (baseline), immediately (acute), and at 1 h, 4 h, and 24 h after CM application.

To record cortical network activity, we used commercially available 60MEA200/30iR-T (Multi Channel Systems (MCS), Reutlingen, Germany) and a 60-channel MEA filter-amplifier (MEA2100-Mini-HS60, MCS, Reutlingen Germany) connected to an interface board (MCS-IFB 3.0 Multiboot Interface Board, MCS, Reutlingen, Germany). All recordings were conducted in an incubator at 37 °C and 5% CO_2_. Raw signals were recorded with a software user interface (MC Experimenter, MCS, Reutlingen, Germany) and analyzed offline (MC Analyzer, MCS, Reutlingen, Germany). All signals were filtered by a second-order Bessel high-pass filter (cut-off at 200 Hz). Spikes represent the spontaneous activity of cortical neurons at respective electrodes, whereas bursts generally indicate network activity [38]. The spikes in the filtered data streams were detected by a negative threshold (-4.5 StDev of the peak-to-peak noise) [39, 40]. The number of spikes within a 5-minute time interval was analyzed. The mean number of spikes per second, the number of bursts per second, various burst features, and timestamps of spikes recorded from individual electrodes were extracted by the MC_Analyzer and analyzed statistically (GraphPad Prism Version 8.0.2, GraphPad Software Inc., San Diego, CA, USA). Complex spike trains were considered as burst activity if they met the following criteria: 20 ms maximum inter-spike interval to start the burst, 10 ms maximum inter-spike interval to end the burst, 10 ms minimum inter-burst interval, 20 ms minimum burst-duration with at least 4 spikes in each burst [40, 41]. Mean values ± SEM were established among equally treated samples. A total of n = 10 electrodes out of 4 batches per group was evaluated. Representative raster plots and voltage traces were generated using custom MATLAB (Mathworks, 2021b) script.

### Real-time quantitative PCR (RT-qPCR)

RNA from cultivated cells was isolated using the GeneUP total RNA mini Kit (Biotechrabbit, Henningsdorf, Germany) according to the manufacturer’s protocol. RNA concentration and purity were evaluated photometrically (FLUOstar Omega, BMG LAB-TECH, Ortenberg, Germany). According to the manufacturer’s recommendations, the total RNA (10 ng) was converted to cDNA by reverse transcription with the QuantiTect Transcription Kit (Qiagen, Hilden, Germany). All primers used in this study were obtained from Biolegio (Nijmegen, The Netherlands). Primer sequences and PCR parameters are listed in Table 1. The samples were amplified and quantified on a LightCycler 96 (Roche, Mannheim, Germany). PCR product integrity was evaluated by melting point analysis and agarose gel electrophoresis. The threshold cycle (CT) was normalized to housekeeping ribosomal protein L13a (RPL13a; ΔCT). Data are depicted as 2^(−ΔΔCT)^. RT-qPCR was performed in technical triplets, and each experiment was conducted in at least biological triplicate. Mean values ± SEM were calculated for all samples.

**Table 1:** Primers and parameters of RT-qPCR

RNA	Sequences forward/reverse 5′–3′	Temperature (°C) step 1/2/3	Duration (s) step 1/2/3	Accession number
AT1a	AGTCCTGTTCCACCCGATCA/ CTTTCTGGGAGGGTTGTGTGA	95/56.0/72	15/15/45	NM_030985.4
AT1b	TCAAGCTGTTCTGTCCCA/ GCTACAACTTCAATCAAACAACAGC	95/56.0/72	15/15/45	NM_031009.2
AT2	ACTGGCTCTTTGGACCTGTG/ GCTTGCCAGGGATTCCTTCT	95/56.0/72	15/15/45	NM_012494.3
S100A10	CACACCTTGATGCGTCCTCT/ GGCAACCGGATGCAAACAAT	95/60.0/72	15/15/45	NM_031114.1
Ki67	TCTTGGCACTCACAGTCCAG/ GCTGGAAGCAAGTGAAGTCC	95/58.0/72	15/15/45	NM_001271366.1
EAAT2	GAGAGAGGCTGCCCGTTAAA/ CTTCCACCTGCTTGGGCATA	95/58.0/72	15/15/45	NM_017215.2
iNOS	GCTTGTCTCTGGGTCCTCTG/ CTCACTGGGACAGCACAGAA	95/59.0/72	15/15/45	NM_012611.3
CD206	AACAAGAATGGTGGGCAGTC/ CCTTTCAGTCCTTTGCAAGC	95/56.0/72	15/15/45	NM_001106123.2
IL-6	CCCAACTTCCAATGCTCTCCT/ AGCACACTAGGTTTGCCGAG	95/57.3/72	15/15/45	
IL-10	GAAAAATTGAACCACCCGGCA/ TTTCCAAGGAGTTGCTCCCG	95/56/72	15/15/45	
Cx43	CTCACGTCCCACGGAGAAAA/ CGCGATCCTTAACGCCTTTG	95/56.0/72	15/15/45	NM_012567.2
RPL13a	TCTCCGAAAGCGGATGAACA/ CAACACCTTGAGGCGTTCCA		15/15/45	NM_173340.2

### Live/dead assay

To evaluate cell survival, dead cells were stained with propidium iodide (Life Technologies, Darmstadt, Germany) while all cells, independently of viability, were counterstained with Hoechst 33342 (Life Technologies, Darmstadt, Germany). Representative pictures were taken using an inverted fluorescence phase-contrast microscope (Keyence BZ-9000E). Five images per well were taken, and both Hoechst-stained and propidium iodide-stained cells were counted manually. The ratio of propidium iodide positives on total cell count provided the proportion of cell death. The experiment was performed in at least triplicate with four wells per condition. The resulting mean values ± SEM were established among equally treated samples.

### Immunocytochemical staining of astrocytes and microglia

Characteristics of astrocytes in culture were determined immunocytochemically to verify the homogeneity of the cultures. Cells were fixed with 4% paraformaldehyde (PFA) and stained against Glia Fibrillary Acidic Protein, clone GA5 (GFAP; mouse monoclonal antibody, dilution 1:500, Cat# MAB360, RRID:AB_11212597, Merck, Darmstadt, Germany) in order to identify astrocytes. To evaluate the expression of the different types of angiotensin II receptors, primary astrocytes were stained with angiotensin II receptor, type 1 antibody (AT1; rabbit polyclonal antibody, dilution 1:500, Cat# SAB3500209, RRID:AB_10638798, Sigma Aldrich, Taufkirchen, Germany), which identifies AT1A and AT1B sharing > 94% sequence homology in rodents, and angiotensin II receptor, type 2 antibody (AT2; rabbit polyclonal antibody, dilution 1:500, ab19134, RRID:AB_2273884, Abcam, Milton, UK), respectively [42, 43]. Anti-S100A10 (polyclonal rabbit antibody, dilution 1:100, Cat# NBP1-89370, RRID:AB_11012229, Novus, Centennial, USA) was used to identify S100A10 on astrocytes. Anti-Myelin Basic Protein (MBP; rabbit polyclonal antibody, dilution 1:100, Cat# ab40390, RRID:AB_1141521, dilution 1:100, Abcam, Milton, UK) was used to stain for oligodendrocytes.

All microglia were stained against the ionized calcium-binding adapter molecule 1 (Iba1; rabbit polyclonal antibody, dilution 1:500, cat. 019-19741, RRID:AB_839504, FUJIFILM WAKO, Osaka, Japan). Microglia were stained for inducible nitric oxide synthase (iNOS) (mouse monoclonal antibody, dilution 1:500, cat. 49999, RRID:AB_881438, Abcam, Milton, UK) to characterize the induction of a pro-inflammatory phenotype. For visualization, fluorescein-labeled anti-mouse IgG or anti-rabbit IgG were used (dilution 1:200, Invitrogen, Karlsruhe, Germany); all cells were additionally counterstained with Hoechst 33342 (Life Technologies, Darmstadt, Germany). Representative images were taken using a digital microscope (Keyence BZ-9000E, Osaka, Japan).

### Adenosine triphosphate (ATP)-assay

According to the manufacturer’s instructions, astrocytes were seeded on a Nunc™ F96 MicroWell™ White Polystyrene Plate (Cat# 236105, Thermo Fisher Scientific, Waltham, USA). ATP concentrations of astrocytes were quantified using an ATP colorimetric/fluorometric assay kit (Abcam, Cat# ab83355, Abcam, Milton, UK). Mean values ± SEM were established among equally treated samples. Each experiment was conducted in at least triplicate.

### Glutamate assay

The glutamate concentration in the media of cultured astrocytes was quantified using a Glutamate Detection Assay Kit (Abcam, Cat# ab83389, Abcam, Milton, UK) following the manufacturer’s instructions. Mean values ± SEM were established among equally treated samples. Each experiment was conducted in at least triplicate.

### Immunofluorescence analysis

Intensity parameters of immunofluorescent staining of S100A10 in GFAP positive astrocytes and iNOS in Iba1 positive microglia, respectively, were analyzed using a custom MATLAB (Mathworks, 2021b) script. In brief, a mask of GFAP or Iba1 positive pixels was established using Otsu’s method. Next, statistical parameters (i.e., mean, median, standard deviation and signal-to-noise ratio) of S100A10 or iNOS intensity values within the derived masks were extracted based on histogram function. Mean values ± SEM were established among equally treated samples. Each experiment was conducted in at least triplicate.

### Data processing and statistical analysis

Raw numerical data were processed with Microsoft Excel (Version 2019, Microsoft Corp., Seattle, WA, USA). Images were edited with ImageJ (v.2.0.0 Fiji) and Adobe Photoshop Elements 2020 (Adobe Inc., San Jose, CA, USA) by adjusting brightness, contrast, and sharpness for each color channel minimally without exerting significant modifications. Figures were created using Adobe Illustrator (Adobe Inc., San Jose, CA, USA).

At least three technical replicates were carried out for the cell culture experiments. Statistical analyses and graphic visualization were performed with GraphPad Prism (Version 8.0.2, GraphPad Software Inc., San Diego, CA, USA). If all variables analyzed met the assumption of normality, a t-test or one-way Analysis of Variance (ANOVA) was conducted to compare multiple groups. ANOVA was followed by Dunnett’s, Tukey’s, or Holm-Sidak’s post-hoc tests with the same software. Repeated measure two-way ANOVA was used for MEA evaluation. In case parameters turned out to be not normally distributed, the non-parametric Mann–Whitney-U test or in case of comparing multiple groups, Kruskal–Wallis test was calculated and followed up by Dunn’s test. A p-value < 0.05 was considered to indicate significance. Statistical significance is denoted as not significant (n.s.), *p < 0.05, **p < 0.01, or ***p < 0.001.

## Results

### Immunocytochemical staining of AT1 and AT2 shows unspecific binding with commercial antibodies

We used commercially available antibodies to stain AT1 (SAB3500209) and AT2 (ab92445), respectively. However, in previous studies, commercially available AT1 and AT2 antibodies have shown lack in specificity [44, 45]. Both receptors had been previously used; within their validation process their use was critically evaluated [46, 47]. During the validation process with HEK293 cells as negative controls we found that both antibodies showed unspecific binding (data not shown). Thusly, we used RT-qPCR to validate the expression of both angiotensin receptors. To exclude significant contamination of our astrocyte culture by other cell entities, we conducted immunocytochemical stainings for GFAP (astrocytes), Iba1 (microglia) and MBP (oligodendrocytes) (suppl. Figure 1A, C). GFAP-positive cells made up a majority of at least 86% and were significantly higher than Iba1-positive cells (8%, suppl. Figure 1B) and MBP-positive cells (0.8%, suppl. Figure 1D).

### Astrocytes express AT1 and AT2

**Fig. 1:**
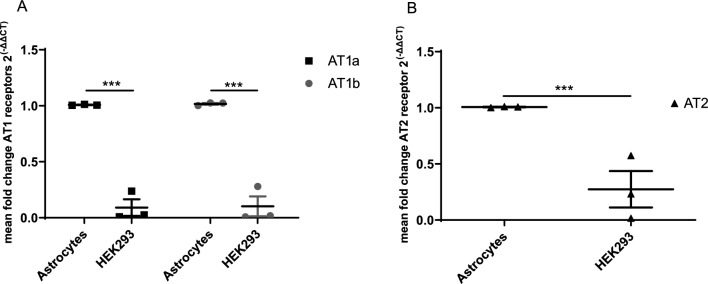
Astrocytes express AT1 and AT2 receptors. *p < 0.05, **p < 0.01, and ***p < 0.001 compared different experimental groups as marked by horizontal bar; graphs depict mean values ± standard error of the mean (SEM). **A** Expression of AT1A and AT1B on primary rat astrocytes (control) compared to HEK293 cells on mRNA-level measured by RT-qPCR. **B** Expression of angiotensin II receptor, type 2 (AT2) on primary rat astrocytes (control) compared to HEK293 cells

In contrast to humans, rodents express two AT1 subtypes with high sequence identity, AT1A and AT1B, and most of the central effects of AT1 stimulation correspond to AT1A activation [48–51]. The expression of AT1A and AT1B on primary rat astrocytes at mRNA-level was determined by RT-qPCR (Fig. 1A), and HEK293 cells served as negative control (mean fold change: AT1A astrocytes = 1.01 vs. AT1A HEK93 = 0.09; n = 3/group, t-test: p < 0.0001; mean fold change: AT1B astrocytes = 1.02 vs. AT1B HEK293 = 0.10; n = 3/group, t-test; p < 0.0001).

The expression of AT2 was determined on mRNA and protein level by RT-qPCR (mean fold change: AT2 astrocytes = 1.01 vs. AT2 HEK93 = 0.274; n = 3/group, t-test: p < 0.0001; Fig. 1B).

### Blockade of AT1 by telmisartan enhances cell survival in OGD-treated astrocytes

**Fig. 2:**
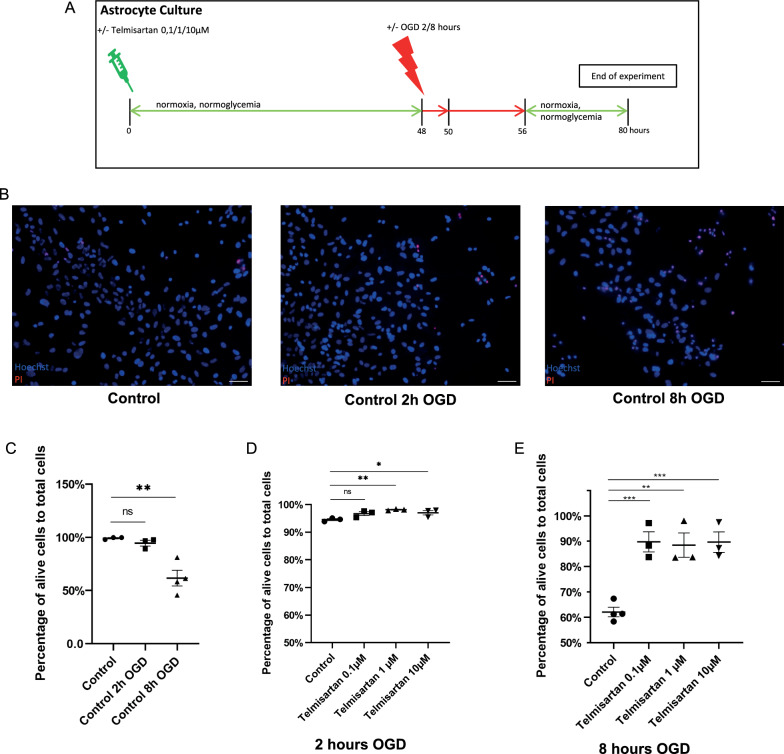
Telmisartan affects survival of astrocytes in an in vitro stroke model. *p < 0.05, **p < 0.01, and ***p < 0.001 compared different experimental groups as marked by horizontal bar; graphs depict mean values ± standard error of the mean (SEM). **A** Experimental timeline. Three to five days after subculturing, astrocytes were left unstimulated or treated with 0.1 µM, 1 µM, or 10 µM telmisartan for 48 h. They were then exposed to 2 h or 8 h of oxygen–glucose deprivation (OGD) before they were allowed to recover in regular culture medium for 24 h. Afterward, the cells were used for further experiments. Unstimulated astrocytes, which were also exposed to 2 h or 8 h of OGD, respectively, served as controls. **B** Representative immunocytochemical staining for cell viability using propidium iodide (PI, red) of untreated astrocytes (Control) and astrocytes treated for 2 and 8 h, respectively, with OGD (Control 2 h OGD and Control 8 h OGD). Hoechst stained all cell nuclei blue; scale bar = 50 µm. **C** The percentage of alive cells to total cells did not change significantly for control cells under unstimulated conditions or 2 h of OGD, but changed significantly after 8 h of OGD. **D** After 2 h of OGD, the percentage of alive cells to total cells increased significantly with the preincubation with 1 µM and 10 µM telmisartan, respectively, compared to cells only subjected to OGD. **E** The percentage of alive cells to total cells changed significantly with the preincubation with telmisartan at different concentrations compared to control cells after 8 h of OGD

To investigate the effects of AT1-inhibition by telmisartan on the survival of primary astrocytes subjected to OGD-induced cell death, cells were preincubated for 48 h with 0.1 µM, 1 µM, and 10 µM telmisartan, before subjecting them to 2 h (acute) and 8 h (prolonged) of OGD with a subsequent reoxygenation time of 24 h (Fig. 2A). To assess the effects of OGD on control cells, cell survival was compared between untreated cells (Control), cells subjected to 2 h of OGD (Control 2 h) and cells subjected to 8 h of OGD (Control 8 h). Cell viability was assessed using Live/Dead assay, staining dead cells with propidium iodide while all cells, independently of viability, were counterstained with Hoechst 33342 (Fig. 2B). 99.2% of control cells survived compared to 94.5% of cells after 2 h of OGD (Control = 99.2% vs. Control 2 h OGD = 94.5%, n = 3/group, ANOVA, Dunnett’s post hoc test, p = 0.895; Fig. 2C). The number of surviving cells was significantly reduced to 62% after 8 h of OGD (Control = 99.2% vs. Control 8 h OGD = 62%, n = 3/group, ANOVA, Dunnett’s post hoc test, p < 0.01; Fig. 2C).

After 2 h of OGD, the pre-stimulation of astrocytes with telmisartan increased cell survival in OGD-treated cells dose-dependently (Fig. 2D): Preincubation with 1 µM telmisartan increased the number of alive cells to total cells to 98.1% and the preincubation with 10 µM telmisartan to 97% compared to 94.5% for OGD-treated control cells (control = 94.5% vs. telmisartan 1 µM = 98.1%, n = 3/group, ANOVA, Dunnett’s post hoc test: p < 0.01; control = 94.5% vs. telmisartan 10 µM = 97%, n = 3/group, ANOVA, Dunnett’s post hoc test: p < 0.05; F (3, 8) = 7.222, p = 0.0115; Fig. 2D). The pre-treatment of OGD-treated astrocytes to 0.1 µM telmisartan did not show significant changes (control = 94.5% vs. telmisartan 0.1 µM = 96.7%, n = 3/group, ANOVA, Dunnett’s post hoc test: p < 0.06, n.s.; Fig. 2D).

After 8 h of OGD, the overall survival rate of astrocytes was significantly affected by the preincubation with telmisartan at different concentrations (control = 62% vs. telmisartan 0.1 µM = 89.8%, n = 3/group, ANOVA, Dunnett’s post hoc test: p < 0.001; control = 62% vs. telmisartan 1 µM = 88.5%, n = 3/group, ANOVA, Dunnett’s post hoc test: p < 0.01; control = 62% vs. telmisartan 10 µM = 89.7%, n = 3/group, ANOVA, Dunnett’s post hoc test: p < 0.001; F (3, 9) = 16.57, p = 0.0005; Fig. 2E).

As merely 62% survival of astrocytes exposed to 8 h of OGD was observed, the benefit of these cells and their conditioned media for further experiments at this time was questionable as the health of actively secreting molecules of astrocytes was likely compromised.

**Fig. 3:**
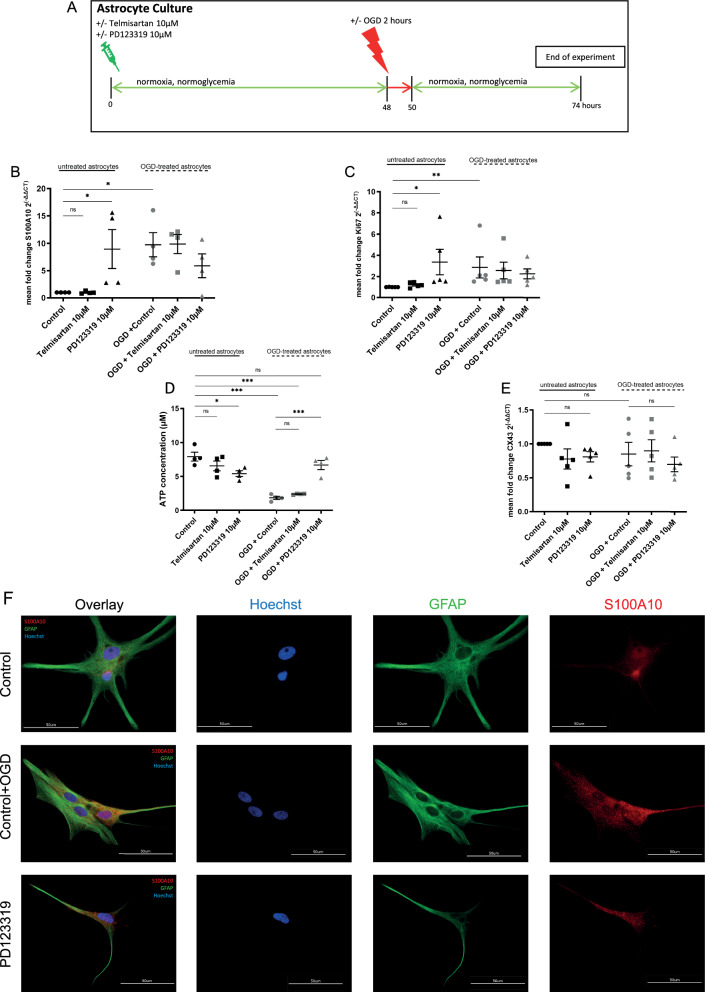
The AT2-blocker PD123319 induces S100A10 expression in astrocytes with increased proliferation whereas Telmisartan does not affect S100A10 or Ki67 expression. *p < 0.05, **p < 0.01, and ***p < 0.001 compared different experimental groups as marked by horizontal bar; graphs depict mean values ± standard error of the mean (SEM). **A** Experimental timeline. Three to five days after subculturing, astrocytes were left unstimulated or treated with 10 µM telmisartan or 10 µM PD123319 for 48 h. They were then exposed to 2 h of oxygen–glucose deprivation (OGD) before they were allowed to recover in regular culture medium for 24 h. Afterward, the cells were used for further experiments. Unstimulated astrocytes, which were exposed to 2 h of OGD, served as controls. **B** S100A10 marker expression in untreated (unstimulated) or OGD-treated (2 h of OGD) astrocytes with or without the additional preincubation with 10 µM telmisartan or 10 µM PD123319. **C** Proliferation rate of untreated (unstimulated) and OGD-treated (2 h OGD) astrocytes, with or without preincubation with 10 µM telmisartan or 10 µM PD123319 over 48 h as assessed by mRNA-ki67 expression. **D** ATP concentration of untreated or OGD-treated astrocytes with or without preincubation with 10 µM telmisartan or 10 µM PD123319 over 48 h. **E** Expression of the connexin Cx43 in untreated or OGD-treated astrocytes with or without preincubation with 10 µM telmisartan and 10 µM PD123319 over 48 h. **F** Representative immunocytochemical stainings of astrocytes with Glia Fibrillary Acidic Protein (GFAP) + (green) and S100A10 + (red) in unstimulated (control), PD123319-preincubated (PD123319), or after 2 h of OGD (OGD + Control). Hoechst stained all cell nuclei blue; scale bars = 50 µm

S100A10 of the S100 protein family has been identified as a marker of astrocytes involved in neuroprotection, promoting neuronal survival and tissue repair [8, 40]. Astrocytes exposed to OGD for 2 h increased expression of S100A10, while telmisartan did not additionally affect the S100A10 marker expression in untreated as well as OGD-treated astrocytes compared to the respective controls (control = 1 vs. telmisartan 10 µM = 1.0; n = 4/group, ANOVA, Holm-Sidak’s post hoc test: n.s.; 9.76-fold for OGD-treated astrocytes (OGD + control) compared to untreated control cells = 1; n = 4/group, ANOVA, Holm-Sidak’s post hoc test: p < 0.05; OGD + control = 9.76 vs. OGD + telmisartan 10 µM = 9.88; n = 4/group, ANOVA, Holm-Sidak’s post hoc test: n.s.; F (5, 18) = 4.178, p = 0.0107 Fig. 3B).

Proliferation rate as measured by Ki67-expression on the mRNA-level was increased in OGD-treated astrocytes exposed to 2 h of OGD compared to untreated control cells (2.86-fold compared to untreated control = 1; n = 5/group, Kruskal–Wallis Test, followed by Dunn’s post hoc test: p < 0.01.; Fig. 3C). The additional treatment with 10 µM telmisartan did not affect the proliferation rate of untreated or OGD-treated astrocytes (Fig. 3C).

### AT2-inhibition induces a proliferating phenotype with increased S100A10 expression

Cells were preincubated 48 h with 10 µM PD123319 before subjecting them to 2 h of OGD with a subsequent reoxygenation time of 24 h to investigate the effects of the selective AT2-inhibitor PD123319 on the characterization of primary astrocytes subjected to OGD (Fig. 3A). The treatment of astrocytes with PD123319 over 48 h resulted in a significant increase in the expression of S100A10 on mRNA-level (8.96-fold compared to untreated control = 1; n = 4/group, ANOVA, Holm-Sidak’s post hoc test: p < 0.05; F (5, 18) = 4.178, p = 0.0107; Fig. 3B). As mentioned above, astrocytes exposed to OGD for 2 h increased expression of S100A10, and PD123319 did not have an additional modulating effect (9.76-fold for OGD-treated astrocytes compared to untreated control cells = 1; n = 4/group, ANOVA, Holm-Sidak’s post hoc test: p < 0.05; PD123319-stimulated astrocytes with OGD = 5.89-fold compared to control = 1; n = 4/group, ANOVA, Holm-Sidak’s post hoc test: n.s.; F (5, 18) = 4.178, p = 0.0107; Fig. 3B). Representative immunocytochemical stainings of astrocytes against S100A10 protein are shown for control-, PD123319-stimulated and OGD-stimulated astrocytes (Fig. 3F). However, intensity measurements showed no significant changes between the three groups (suppl. Figure 3A).

Furthermore, coinciding with the elevated expression of S100A10, the proliferation rate of resting astrocytes exposed to PD123319 was significantly increased, as assessed by Ki67 expression (3.37-fold compared to control = 1; n = 5/group, Kruskal–Wallis Test, followed by Dunn’s post hoc test: p < 0.05; Kruskal–Wallis statistic 19.18; Fig. 3C).

As an essential metabolite, ATP regulates multiple intracellular reactions and can consequently serve as a marker for energy levels [52]. The total ATP-concentration of astrocytes was decreased upon AT2 blockade by PD123319 (5.4 µM) compared to unstimulated astrocytes (7.9 µM; n = 4/group, ANOVA, Tukey’s post hoc test: p < 0.05, Fig. 3D). Interestingly, OGD-stimulation resulted in an even lower ATP-concentration (1.9 µM), and this was counteracted by additional preincubation with PD123319 (6.7 µM) (OGD + control vs. OGD + PD123319 10 µM; n = 4/group, ANOVA, Tukey’s post hoc test: p = 0.0001; Fig. 3D). Of note, blocking AT1 by telmisartan did not affect ATP-concentrations of untreated or OGD-treated astrocytes (control = 7.9 µM vs. telmisartan = 6.6 µM; n = 4/group, ANOVA, Tukey’s post hoc test: n.s.; OGD + Control = 1.9 µM vs. OGD + telmisartan = 2.4 µM; n = 4/group, ANOVA, Tukey’s post hoc test: n.s.; Fig. 3D).

Astrocytes create a highly interconnected network within the CNS via gap junctions or hemichannels made from connexins of the subtypes Cx30 and Cx43, which allows a rapid intercellular exchange of ions and metabolites such as ATP [53]. Though we could determine the expression of Cx43 in astrocytes, its expression remained unaffected by telmisartan-, PD123319- and/or OGD-treatment (n = 5/group, ANOVA, Tukey’s post hoc test, F (5, 24) = 0.6766, p = 0.6453; Fig. 3E).

### The conditioned medium of astrocytes after AT1 and AT2 blockade differentially modulates the inflammatory phenotype of microglia

**Fig. 4:**
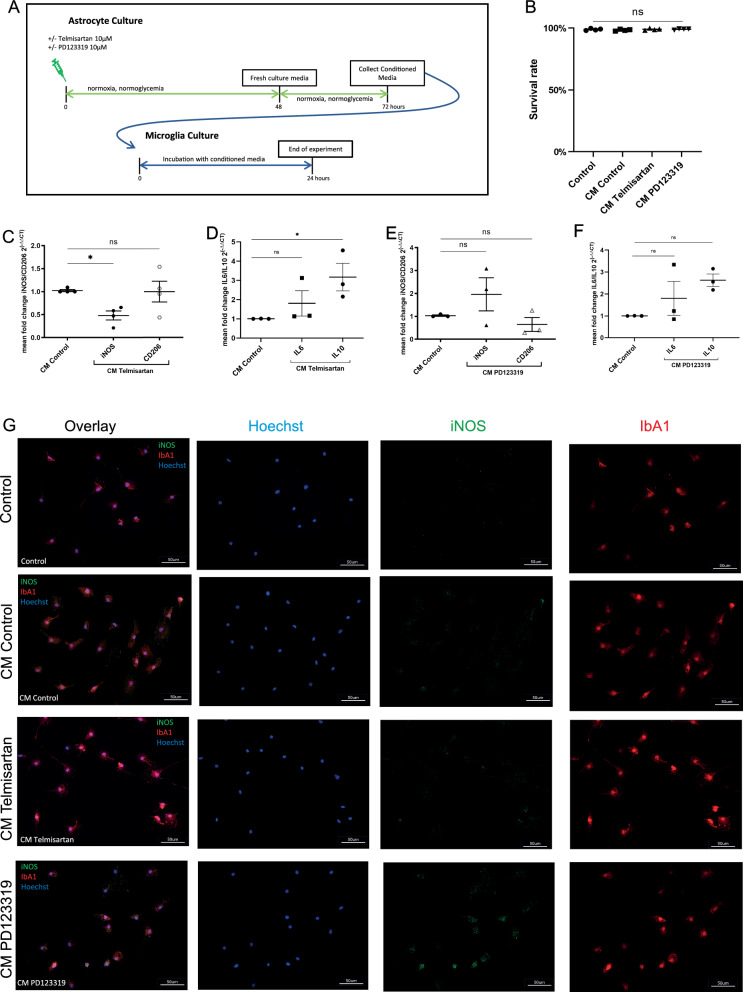
The incubation with angiotensin receptor blockers (ARBs) of astrocytes affects the inflammatory phenotype of microglia. *p < 0.05, **p < 0.01, and ***p < 0.001 compared different experimental groups as marked by horizontal bar; graphs depict mean values ± standard error of the mean (SEM). **A** Experimental timeline. Three to five days after subculturing, astrocytes were left unstimulated (control), treated with 10 µM PD123319 or 10 µM telmisartan for 48 h. Next, fresh culture medium was added, and the cells were allowed to recover in regular culture medium over 24 h. This so-called conditioned medium (CM) of astrocytes was consecutively collected for further experiments. Following 24 h after seeding in subcultures, microglia were exposed to CM from resting astrocytes (CM Control), CM from astrocytes stimulated with 10 µM PD123319 (CM PD123319) or CM from astrocytes stimulated with 10 µM telmisartan (CM Telmisartan) over 24 h. Consecutively, RNA was isolated or microglia were fixed with 4% paraformaldehyde (PFA) for immunocytochemical staining. **B** Microglial viability was not compromised through the exposure to CM Control (98.4% survival rate), CM Telmisartan (99% survival rate) or CM PD123319 (99.1% survival rate) compared to untreated control cells (98.9% survival rate). **C** Expression of the pro-inflammatory microglia marker inducible nitric oxide-synthetase (iNOS) and the anti-inflammatory microglia marker CD206 between CM Control and CM Telmisartan. **D** Expression of the pro-inflammatory microglia marker IL-6 and the anti-inflammatory microglia marker IL-10 between CM Control and CM Telmisartan. **E** Expression of the pro-inflammatory microglia marker iNOS and the anti-inflammatory microglia marker CD206 between CM Control and CM PD123319. **F** Expression of the pro-inflammatory microglia marker IL-6 and the anti-inflammatory microglia marker IL-10 between CM Control and CM PD123319. **G** Representative immunocytochemical stainings of microglia with IbA1 + (red) and iNOS + (green) in unstimulated (control), CM control-preincubated (CM Control), CM telmisartan-preincubated (CM Telmisartan) or PD123319-preincubated (CM PD123319) conditions. Hoechst stained all cell nuclei blue; scale bars = 50 µm

To investigate the modulatory effect of astrocytes on microglia via secretory molecules, microglia were exposed to conditioned medium (CM) from untreated astrocytes (CM Control), CM from astrocytes treated with 10 µM PD123319 (CM PD123319), or CM from astrocytes treated with 10 µM telmisartan (CM Telmisartan) for 24 h (Fig. 4A). Consecutively, microglia were used for either mRNA-extraction or fixation for immunocytochemistry. Survival rate of untreated microglia was 98.9% and treatment with CM Control (98.4% survival rate), CM Telmisartan (99% survival rate) or CM PD123319 (99.1% survival rate) did not cause any changes in viability compared to untreated microglia (n = 4/group, ANOVA followed by Dunnett’s multiple comparisons test, F (3, 12) = 0.9126, p = 0.4639; Fig. 4B).

CM Telmisartan significantly decreased the expression of the pro-inflammatory microglia marker iNOS (0.48-fold compared to cells exposed to CM control = 1; n = 4/group, AONVA followed by Dunnett’s post hoc test: p < 0.05, Fig. 4C), but did not affect the expression of the anti-inflammatory marker CD206 (Fig. 4C).

Interestingly, CM Telmisartan significantly increased the expression of the anti-inflammatory marker interleukin 10 (IL-10) by 3.17-fold in microglia compared to microglia exposed to CM Control; no significant differences were observed between CM Telmisartan and CM Control for the pro-inflammatory marker interleukin 6 (IL-6) (CM Control = 1 vs. CM Telmisartan (IL-10) = 3.17, n = 3/group, Kruskal–Wallis test followed by Dunn’s post hoc test; p < 0.05; CM Control = 1 vs. CM Telmisartan (IL-6) = 1.81, Kruskal–Wallis test followed by Dunn’s post hoc test; n.s.; Fig. 4D).

The exposure to CM PD123319 showed increased expression of pro-inflammatory markers iNOS and IL-6 by trend (CM control = 1 vs. CM PD123319 (iNOS) = 1.96; n = 4/group, ANOVA followed by Dunnett’s post hoc test: n.s.; CM Control = 1 vs. CM PD123319 (IL-6) = 1.80, n = 3/group, Kruskal–Wallis test followed by Dunn’s post hoc test: n.s.; Fig. 4E, F). Interestingly, the anti-inflammatory marker CD206 showed slight decrease while IL-10 was increased by trend on microglia (CM control = 1 vs. CM PD123319 (CD206) = 0.65; n = 4/group, ANOVA followed by Dunnett’s post hoc test: n.s.; CM Control = 1 vs. CM PD123319 (IL-10) = 2.63, n = 3/group, Kruskal–Wallis test followed by Dunn’s post hoc test: n.s.; Fig. 4E, F).

Representative immunocytochemical stainings for iNOS are shown in Fig. 4G (Fig. 4G). Intensity measurements for iNOS of the different groups revealed a significant increase for CM PD123319 compared to CM Control as well as a decrease by trend for CM Telmisartan compared to CM Control (CM Control = 35.4 vs. CM PD123319 = 68.0; n = 4/group, ANOVA followed by Dunnett’s post hoc test: p = 0.0028; CM Control = 35.4 vs. CM Telmisartan = 17.14; n = 4/group, ANOVA followed by Dunnett’s post hoc test; n.s.; suppl. Figure 3B).

### The conditioned medium of astrocytes after AT1 and AT2 blockade modulates functional neuronal activity

**Fig. 5:**
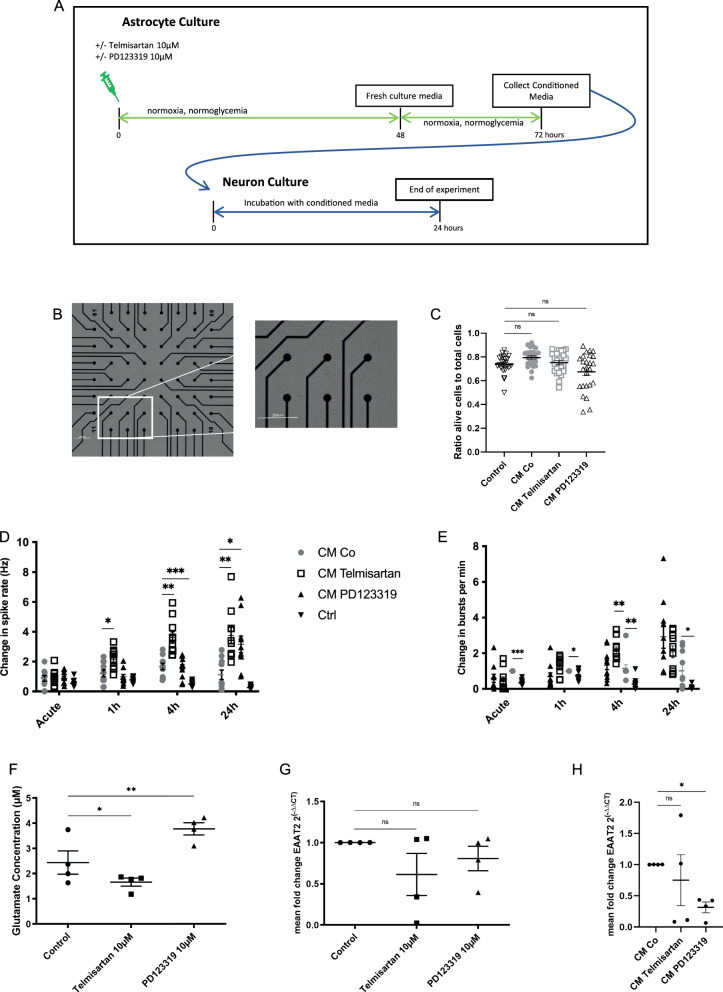
ARBs affect the crosstalk between astrocytes and cortical neurons. *p < 0.05, **p < 0.01, and ***p < 0.001 compared different experimental groups as marked by horizontal bar; graphs depict mean values ± standard error of the mean (SEM). **A** Experimental timeline. Three to five days after subculturing, astrocytes were left unstimulated (control), treated with 10 µM PD123319 or 10 µM telmisartan for 48 h. Next, fresh culture medium was added, and the cells were allowed to recover in regular culture medium over 24 h. This so-called conditioned medium (CM) of astrocytes was consecutively collected for further experiments. For the experiments, half of the neurobasal media was removed and replaced with CM of unstimulated astrocytes (CM Co), 10 µM telmisartan- (CM Telmisartan) and 10 µM PD123319- (CM PD123319) stimulated astrocytes. A naïve control group (Ctrl) received the same volume of neurobasal media. **B** Representative brightfield image of cortical neurons after 15 days of culture on a 60MEA200/30iR-T (MCS, Reutlingen, Germany) with additional focus on six representative electrodes; scale bars = 200 µM. **C** The ratio of alive cells to total cells did not change significantly for neurons exposed to CM Co, CM Telmisartan, or CM PD123319 compared to untreated control cells. **D** Changes in spike rate (Hz) were studied in relation to baseline measurements between the different treatment groups at different time points: Measurements were performed before addition of CM (baseline), immediately (acute), and at 1 h, 4 h, and 24 h after application of CM of unstimulated astrocytes (CM Co), 10 µM telmisartan- (CM Telmisartan), and 10 µM PD123319- (CM PD123319) stimulated astrocytes for 5 min, respectively. A naïve control group (Ctrl) received the same volume of neurobasal media. **E** Changes in burst rate per min were studied in relation to baseline measurements between the different treatment groups at different time points. Data are shown as mean ± SEM. For the MEA data, n = 40 networks were used. **F** Glutamate concentration of untreated resting, telmisartan-stimulated, and PD123319-stimulated astrocytes after 48 h of incubation. **G** Expression of the excitatory amino acid transporter 2 (EAAT2) in untreated resting, telmisartan-stimulated, and PD123319-stimulated astrocytes after 48 h of incubation. **H** Expression of EAAT2 in CM Control, CM Telmisartan, and CM PD123319-stimulated neurons after 48 h of incubation

Next, we investigated the effect of differentially preincubated astrocytes on the functionality of neuronal networks grown on MEAs (Fig. 5A, B).

Changes in spike rate (Hz) and burst rate (per minute) were studied in relation to baseline measurements between the different treatment groups at different time points. Statistical testing was conducted between CM Co and the respective treatment groups (CM Telmisartan or CM PD123319) or untreated control group (Ctrl) at the different time points (acute, 1 h, 4 h, and 24 h) using repeated measure two-way ANOVA followed by Dunnett’s multiple comparisons test. A total of n = 10 electrodes out of 4 batches per group was evaluated. Representative raster plots and voltage traces can be found under supplemental Fig. 4.

Treatment with CM Co, CM Telmisartan, or CM PD123319 did not cause any changes in viability compared to untreated control neuronal cells (n = 25/group, ANOVA, followed by Dunnett’s multiple comparisons test, F (3, 96) = 5.542, p = 0.0015; Fig. 5C).

The CM of untreated astrocytes (CM Co) resulted in only slight changes in spike activity and burst rate changes compared to the untreated control group.

Interestingly, the CM Telmisartan group demonstrated a steady increase both in spike activity and burst rate, starting at 1 h after stimulation, compared to neuronal activity when exposed to CM Co (spike activity CM Co = 1.221 vs. CM Telmisartan = 2.168; n = 10/group, two-way repeated measurement ANOVA, Dunnett’s post hoc test: p < 0.05; interaction F (9, 108) = 10.67, p < 0.0001; time F (1,819, 65,48) = 23.57, p < 0.0001; column factor F (3, 36) = 24,26, p < 0.0001, Fig. 5D; burst rate CM Co = 1.0 vs. CM Telmisartan = 1.328; n = 10/group, two-way repeated measurement ANOVA, Dunnett’s post hoc test: p = 0.1613; interaction F (9, 108) = 11.60, P < 0.0001; time F (1.562, 56.24) = 17.20, p < 0.0001; column factor F (3, 36) = 7.877, p = 0.0004, Fig. 5E) and peaking at 4 h (spike activity CM Co = 1.672 vs. CM Telmisartan = 3.624; n = 10/group, two-way repeated measurement ANOVA, Dunnett’s post hoc test: p < 0.01, Fig. 5D; burst rate CM Co = 1.147 vs. CM Telmisartan = 2.151; n = 10/group, two-way repeated measurement ANOVA, Dunnett’s post hoc test: p < 0.01, Fig. 5E). This increase of spike activity and burst rate remained unchanged even after 24 h (spike activity CM Co = 1.119 vs. CM Telmisartan = 3.763; n = 10/group, two-way repeated measurement ANOVA, Dunnett’s post hoc test: p < 0.01, Fig. 5D; burst rate CM Co = 1.015 vs. CM Telmisartan = 2.075; n = 10/group, two-way repeated measurement ANOVA, Dunnett’s post hoc test: p = 0.090, Fig. 5E). By comparison, the CM PD123319 group’s spike activity (CM Co = 1.672 vs. CM PD123319 = 1.576; n = 10/group, two-way repeated measurement ANOVA, Dunnett’s post hoc test: p < 0.001; Fig. 5D) and burst rate (CM Co = 1.147 vs. CM PD123319 = 1.337; n = 10/group, two-way repeated measurement ANOVA, Dunnett’s post hoc test: p = 0.897; Fig. 5E) started to rise with a delay after 4 h compared to CM Co, reaching its peak after 24 h (spike activity CM Co = 1.119 vs. CM PD123319 = 3.160; n = 10/group, two-way repeated measurement ANOVA, Dunnett’s post hoc test: p < 0.05, Fig. 5D; burst rate CM Co = 1.015 vs. CM PD123319 = 2.928; n = 10/group, two-way repeated measurement ANOVA, Dunnett’s post hoc test: n.s., Fig. 5E).

We examined the glutamate concentration in their conditioned medium to identify potential modifying molecules secreted by astrocytes. Exposure to telmisartan resulted in a decrease of the glutamate concentration in the conditioned medium of astrocytes (1.27 µM compared to untreated cells = 2.49 µM; n = 5/group, ANOVA, Dunnett’s post hoc test: p < 0.05; F (2, 12) = 25.54, p < 0.0001; Fig. 5F), while PD123319-treatment led to an increase of the glutamate concentration (4.31 µM compared to untreated cells = 2.49 µM; n = 5/group, ANOVA, Dunnett’s post hoc test: p < 0.01; Fig. 5F). Next, we examined whether the changes in glutamate concentration were affected by mRNA-expression of the excitatory amino acid transporter 2 (EAAT2) as the most frequently expressed glutamate transporter for reuptake on astrocytes [54–56]. Interestingly, treatment with both angiotensin receptor blockers did not change the expression of EAAT2 on astrocytes (telmisartan 10 µM = 0.61 vs. control = 1.0; n = 4/group, ANOVA, Tukey’s post hoc test: n.s.; PD123319 10 µM = 0.80 vs. control = 1.0; n = 4/group, ANOVA, Tukey’s post hoc test: n.s.; F (2, 9) = 1.284; p = 0.3231; Fig. 5G). As EAAT2 is also expressed by neurons [56], we examined the mRNA-expression of EAAT2 on neurons in order to elucidate whether these cells contribute to changes in neuronal activity. CM Telmisartan did not affect EAAT2 mRNA-expression on neurons compared to CM Co-treated neurons; however, CM PD123319 led to a significant decrease in EAAT2 mRNA-expression (CM Control = 1 vs. CM Telmisartan = 0.75; n = 4/group; MWU; n.s.; CM Control = 1 vs. CM PD123319 = 0.32; n = 4/group, MWU; p < 0.05; Fig. 5H).

## Discussion

In the present study, we observed a modulation of untreated and OGD-treated astrocytes by the ARBs telmisartan (AT1-inhibition) and PD123319 (AT2-inhibition). The survival of astrocytes after exposure to 8 h of OGD is compromised (cf. Fig. 2C). In our study, AT1-inhibition significantly protected astrocytes against short and prolonged OGD exposure (cf. Fig. 2D, E). This finding aligns with previous observations made in primary neuron-astrocyte cocultures exposed to 90 min of OGD [35]. Corroborating those results, pre-treatment with telmisartan for 7 days was previously reported to reduce cerebral infarct volumes in rats by reducing cytosolic phospholipase A_2_ (cPLA_2_) protein expression in the peri-infarct cortex. Histopathologically, neurons in the peri-infarct cortical regions of telmisartan-pretreated rats appeared normal, while neurons of vehicle-pretreated rats showed acute ischemic changes [57]. However, those findings had not yet been linked to the role of astrocytes. Our data indicates that AT1-inhibition by sartans affects astrocytes and enhances their survival, especially in ischemic conditions.

We show for the first time that AT2-inihibition modulates the astrocytic phenotype by increasing S100A10 expression (cf. Fig. 3B). Localized predominantly within the cytoplasm of cells, the marker S100A10 of the S100 protein family associated with a protective astrocyte phenotype is essential in maintaining proliferation, cell differentiation, and cell cycle progression [8, 58]. Increased S100A10 expression coincided with increase in the proliferation rate of AT2-inhibited astrocytes compared to unstimulated astrocytes (cf. Fig. 3C). Similar observations on AT2-mediated proliferation have previously been made in rat pheochromocytoma PC12W cells, rat coronary endothelial cells, and CRC cells, in which the stimulation of AT2 by agonists led to a decrease in proliferation [34, 59–61]. We conclude that AT2 signaling might play a modulating role in the phenotype and proliferation capacity of astrocytes.

Similarly, we observed increased S100A10-expression and enhanced proliferation in astrocytes exposed to OGD (cf. Fig. 3B, C). This observation is in line with previous studies, showing cerebral ischemia to induce S100A10-positive reactive astrocytes revealed by transcriptomics [7, 8]. Interestingly, there was no additive or synergistic effect on S100A10 expression or proliferation rate by OGD plus AT2-inhibition compared to OGD or AT2-inhibition alone (cf. Fig. 3B, C). However, further investigations are needed to decipher the respective pathway leading to the enhancement of this astrocytic profile and proliferation capacity under the influence of the ischemic condition or the blockade of AT2.

As the principal homeostatic cells of the CNS, astrocytes are integrated into neural networks, affecting their surrounding cells’ morphological and functional plasticity. This way, the differential activation of astrocytes can define pathological progression in the interaction with other cell entities [8, 40].

As the innate immune cells of the CNS**,** microglia display a dynamic, context-dependent phenotype expression that is affected by the local environment [29, 62]. We show that the CM of telmisartan-treated astrocytes attenuated the expression of the pro-inflammatory marker iNOS on microglia while increasing the expression of the anti-inflammatory marker IL-10 on microglia (cf. Figs. 4C, D). With telmisartan increasing survival of OGD-treated astrocytes (cf. Fig. 2C, D) our results show a phenotype of astrocytes independently of S100A10 mRNA-expression (cf. Fig. 3B), that modulates microglia with its CM towards an anti-inflammatory expression profile.

This finding supports the notion that both astrocytic phenotypes depict a spectrum of respective cell activation and can be modulated diversely [10]. As one of the major excitatory cerebral neurotransmitters, glutamate has also been associated at higher concentrations with several acute and chronic brain diseases including cerebral ischemia [51, 55]. We show that ARBs differentially affect the glutamate concentration of astrocytes by reducing the secreted glutamate concentration through telmisartan (cf. Fig. 5F). This finding is in line with previous examinations on neuron-astrocyte co-cultures, showing a decreased glutamate release by pre-treatment with losartan or telmisartan in an in vitro stroke model [35]. We conclude that by reducing secreted glutamate of astrocytes through AT1-inhibition, microglia are modulated towards an anti-inflammatory phenotype.

Examining the effects of AT2-inhibition in astrocytes in their consecutive interaction with microglia, we were able to observe a shift of microglia towards a rather proinflammatory phenotype as suggested by the slightly increased expression of proinflammatory markers iNOS and IL-6 with anti-inflammatory components as suggested by the slight increase of IL-10 (cf. Fig. 4E, F). Simultaneously, we also observed an increased glutamate concentration in astrocytes upon AT2-inhibition (cf. Fig. 5F). As mentioned in the previous paragraph, glutamate has been associated at higher concentrations with several acute and chronic brain diseases [51, 55]. We suggest that the induced phenotypical change in microglia through AT2-inhibited astrocytes is mediated by glutamate.

Interestingly, the ATP-concentration in AT2-inhibited astrocytes decreased compared to untreated astrocytes, whereas AT2-inhibition led to the contrary effect in OGD-treated astrocytes (cf. Fig. 3D).

Recently, astrocyte subsets were identified as defined by their function and molecular signatures [63]. This, in turn, results in at least two sources of astrocyte heterogeneity: developmentally induced astrocytes (DIAs) and stimulus-induced astrocytes (SIAs) [63]. Lee et al. argue that the addition of astrocyte developmental heterogeneity may induce multiple SIA subsets through the same stimulus. The authors conclude that neurotransmitters, cytokines, and environmental factors can induce SIAs from various DIAs. In this context, previously identified disease-associated astrocytes (DAAs) are one example of SIAs [63–65]. These DIA subsets may converge on a single molecular phenotype induced by disease-related stimuli to DAAs [63]. However, SIAs identified as DAAs may contribute to disease pathogenesis depending on location and disease phase, meaning that astrocyte subsets limiting disease pathogenesis in one neurological disorder may be detrimental in another [63].

Our results corroborate these novel discoveries: despite showing increased S100A10 expression, suggestive of a protective phenotype, astrocytes exposed to AT2-inhibition shift microglia towards a predominantly proinflammatory phenotype. Consequently, we hypothesize a specific stimulus induced astrocyte through AT2-inhibition. This is in line with previous studies, which have shown that AT2-inhibition results in proinflammatory effects of astrocytes in diseases such as Parkinson’s [25].

In comparison, AT1-inhibition resulted in no changes to S100A10-/Ki67-expression or ATP-metabolism in astrocytes but affected the phenotype of microglia, adding to an astrocytic phenotype with consecutive protective properties without changes in the respective expression of the aforementioned molecules.

So far, only a few studies have investigated the interactions between astrocytes and neuronal network activity in vitro [8, 40]. In a recent study examining pluripotent stem cell-derived astrocytes in the interaction with human neurons, the co-stimulation of these astrocytes with IL-1β and TNF-α resulted in an inflammatory reactive phenotype with neurosupportive characteristics including increased neuronal activity observed in MEAs [40].

Sustained or increased neuronal activity has been regarded as a surrogate of preserved or protected network activity [40]. Astrocytes enhance synaptic interconnectivity and the formation and stability of excitatory synapses [66, 67]. We observed increased spontaneous neuronal (spike activity) and functional network activity (burst rate) after treating neurons with the CM of telmisartan-stimulated astrocytes compared to control cells. Neuronal activity steadily increased and was maintained even at the 24 h time point (cf. Fig. 5D, E). Apart from being a major excitatory cerebral neurotransmitter glutamate also acts as an active signal molecule for astrocytes [55]. Our data suggest that ARBs do differentially affect the glutamate concentration of astrocytes by reducing the secreted glutamate concentration through telmisartan (cf. Fig. 5F), in line with previous examinations on neuron-astrocyte co-cultures [35].Conversely, it has been shown that glutamate released from astrocytes stimulates neuronal presynaptic *N*-methyl-d-aspartate (NMDA) receptors or group I metabotropic glutamate receptors (mGluRs), increasing neuronal excitation [68]. The expression of the most frequently expressed glutamate transporter EAAT2 on astrocytes (cf. Fig. 5G) and neurons (cf. Fig. 5H) was not affected by telmisartan.

On the other hand, high neural activity has also recently shown to accelerate the decline of cognitive plasticity [69]. The incubation with CM of PD123319-stimulated astrocytes resulted also in an increase in neuronal network activity, reaching a spike activity and burst rate similar to CM Telmisartan (cf. Fig. 5D, E). We suggest that this is a result of the increased concentration of the excitatory molecule glutamate observed in CM PD123319 (cf. Fig. 5F). We could also show that CM PD123319 of astrocytes leads to a decrease of EAAT2-mRNA-expression on neurons (cf. Fig. 5H). Increased EAAT2-expression on neurons has been associated with neuroprotection as it positively affects glutamate clearance [70]. All in all, our data suggest that the increased glutamate concentration of CM PD123319 affects neurons negatively through increased glutamate expression, resulting in increased neural activity. In order to further comprehend these observed effects, additional long-term and detailed neurophysiological studies are required.

A limitation of this study is that the effects of other, albeit lesser expressed, angiotensin receptors such as MasR and ACE2 were not evaluated on astrocytes. For instance, MasR and ACE2 have shown to enhance vasodilatation and within the brain anti-inflammatory properties, cognition, and cell survival [16, 17, 71].

Future studies are warranted to characterize further the specific mechanisms of astrocytes on both microglia and neurons. These findings could help us further understand the functionality of astrocytes and their regulatory mechanism on other cell entities, potentially identifying new therapeutic options.

## Conclusion

Blockade of AT1 and AT2 differentially modulates the activity, functionality, and CM of primary astrocytes. Moreover, treatment with ARBs differentially altered the interaction of astrocytes both with microglia and neuronal networks. Altogether, positive CNS-effects of telmisartan and PD1239319 may potentially be exploited in human stroke. A deeper mechanistic understanding of their action may eventually result in a novel therapeutic approach for the chronic stage of cerebral ischemia.

### Supplementary Information

Additional file 1: Figure S1.

Additional file 2: Figure S2.

Additional file 3: Figure S3.

Additional file 4: Figure S4.

## Data Availability

The data that support the findings of this study are available from the corresponding author on reasonable request.

## Abbreviations

ANOVA: One-way analysis of variance
ARBs: Angiotensin receptor blockers
AT1: Angiotensin II receptor, type 1
AT2: Angiotensin II receptor, type 2
CM: Conditioned medium
CNS: Central nervous system
EAAT2: Excitatory amino acid transporter 2
GFAP: Glia fibrillary acidic protein
IL-6: Interleukin 6
IL-10: Interleukin 10
iNOS: Inducible nitric oxide synthase
MEA: Microelectrode array
OGD: Oxygen–glucose deprivation
RAAS: Renin–angiotensin–aldosterone system
RT-qPCR: Real-time quantitative PCR

## References

[1] Kim JH, Afridi R, Cho E, Yoon JH, Lim YH, Lee HW (2022). Soluble ANPEP released from human astrocytes as a positive regulator of microglial activation and neuroinflammation: brain renin–angiotensin system in astrocyte-microglia crosstalk. Mol Cell Proteomics.

[2] Sofroniew MV (2009). Molecular dissection of reactive astrogliosis and glial scar formation. Trends Neurosci.

[3] Vay SU, Olschewski DN, Petereit H, Lange F, Nazarzadeh N, Gross E (2021). Osteopontin regulates proliferation, migration, and survival of astrocytes depending on their activation phenotype. J Neurosci Res.

[4] Guttenplan KA, Weigel MK, Prakash P, Wijewardhane PR, Hasel P, Rufen-Blanchette U (2021). Neurotoxic reactive astrocytes induce cell death via saturated lipids. Nature.

[5] Liddelow SA, Barres BA (2017). Reactive astrocytes: production, function, and therapeutic potential. Immunity.

[6] Yin X, Feng L, Ma D, Yin P, Wang X, Hou S (2018). Roles of astrocytic connexin-43, hemichannels, and gap junctions in oxygen-glucose deprivation/reperfusion injury induced neuroinflammation and the possible regulatory mechanisms of salvianolic acid B and carbenoxolone. J Neuroinflammation.

[7] Zamanian JL, Xu L, Foo LC, Nouri N, Zhou L, Giffard RG (2012). Genomic analysis of reactive astrogliosis. J Neurosci.

[8] Liddelow SA, Guttenplan KA, Clarke LE, Bennett FC, Bohlen CJ, Schirmer L (2017). Neurotoxic reactive astrocytes are induced by activated microglia. Nature.

[9] Linnerbauer M, Wheeler MA, Quintana FJ (2020). Astrocyte crosstalk in CNS inflammation. Neuron.

[10] Escartin C, Galea E, Lakatos A, O’Callaghan JP, Petzold GC, Serrano-Pozo A (2021). Reactive astrocyte nomenclature, definitions, and future directions. Nat Neurosci.

[11] O’Connor AT, Clark MA (2018). Astrocytes and the renin angiotensin system: relevance in disease pathogenesis. Neurochem Res.

[12] Phillips IM (1987). Functions of angiotensin in the central nervous system. Annu Rev Physiol.

[13] Guimond MO, Gallo-Payet N. The angiotensin II type 2 receptor in brain functions: an update. Int J Hypertens. 2012;2012. 10.1155/2012/351758 PMC3540774 23320146

[14] Dinh DT, Fraumana G, Johnston CI, Fabiani ME (2001). Angiotensin receptors: distribution, signalling and function. Clin Sci (Lond).

[15] Bakris G (2010). Are there effects of renin–angiotensin system antagonists beyond blood pressure control?. Am J Cardiol.

[16] Jackson L, Eldahshan W, Fagan SC, Ergul A. Within the brain: the renin angiotensin system. Int J Mol Sci. 2018;19. 10.3390/ijms19030876 PMC5877737 29543776

[17] Labandeira-Garcia JL, Rodríguez-Perez AI, Garrido-Gil P, Rodriguez-Pallares J, Lanciego JL, Guerra MJ (2017). Brain renin–angiotensin system and microglial polarization: implications for aging and neurodegeneration. Front Aging Neurosci..

[18] Mckinley MJ, Albiston AL, Allen AM, Mathai ML, May CN, Mcallen RM (2003). The brain renin–angiotensin system: location and physiological roles. Int J Biochem Cell Biol.

[19] Liu G, Hosomi N, Hitomi H, Pelisch N, Fu H, Masugata H (2011). Angiotensin II induces human astrocyte senescence through reactive oxygen species production. Hypertens Res.

[20] Biancardi VC, Son SJ, Ahmadi S, Filosa JA, Stern JE (2014). Circulating angiotensin II gains access to the hypothalamus and brain stem during hypertension via breakdown of the blood–brain barrier. Hypertension.

[21] Milsted A, Barnaf BP, Ransohoff RM, Bridget Brosnihan K, Ferrario CM (1990). Astrocyte cultures derived from human brain tissue express angiotensinogen mRNA (gene expression/renin–angiotensin system/central nervous system/angiotensin). Proc Natl Acad Sci USA.

[22] Stornetta RL, Hawelu-Johnson CL, Guyenet PG, Lynch KR (1979). Astrocytes synthesize angiotensinogen in brain. Science.

[23] Garrido-Gil P, Valenzuela R, Villar-Cheda B, Lanciego JL, Labandeira-Garcia JL (2013). Expression of angiotensinogen and receptors for angiotensin and prorenin in the monkey and human substantia nigra: an intracellular renin–angiotensin system in the nigra. Brain Struct Funct.

[24] Occhieppo VB, Basmadjian OM, Marchese NA, Silvero CMJ, Rodríguez A, Armonelli S (2021). AT1-R is involved in the development of long-lasting, region-dependent and oxidative stress-independent astrocyte morphological alterations induced by Ketamine. Eur J Neurosci.

[25] Dominguez-Meijide A, Rodriguez-Perez AI, Diaz-Ruiz C, Guerra MJ, Labandeira-Garcia JL (2017). Dopamine modulates astroglial and microglial activity via glial renin-angiotensin system in cultures. Brain Behav Immun.

[26] Hong S, JianCheng H, JiaWen W, ShuQin Z, GuiLian Z, HaiQin W (2019). Losartan inhibits development of spontaneous recurrent seizures by preventing astrocyte activation and attenuating blood–brain barrier permeability following pilocarpine-induced status epilepticus. Brain Res Bull.

[27] Boily M, Li L, Vallerand D, Girouard H. Angiotensin II disrupts neurovascular coupling by potentiating calcium increases in astrocytic endfeet. J Am Heart Assoc. 2021;10. 10.1161/JAHA.120.020608 PMC8649296 34459216

[28] Dhanachandra Singh K, Karnik SS. Angiotensin receptors: structure, function, signaling and clinical applications. J Cell Signal. 2017;01. 10.4172/jcs.1000111 PMC4976824 27512731

[29] Rabenstein M, Vay SU, Blaschke S, Walter HL, Ladwig A, Fink GR (2020). Crosstalk between stressed brain cells: direct and indirect effects of ischemia and aglycemia on microglia. J Neuroinflammation.

[30] Stangier J, Su CAPF, Roth W (2000). Pharmacokinetics of orally and intravenously administered telmisartan in healthy young and elderly volunteers and in hypertensive patients. J Int Med Res.

[31] Hazlewood RJ, Chen Q, Clark FK, Kuchtey J, Kuchtey RW (2018). Differential effects of angiotensin II type I receptor blockers on reducing intraocular pressure and TGFβ signaling in the mouse retina. PLoS ONE.

[32] Israili ZH (2000). Clinical pharmacokinetics of angiotensin II (AT1) receptor blockers in hypertension. J Hum Hypertens.

[33] Puddefoot JR, Udeozo UKI, Barker S, Vinson GP (2006). The role of angiotensin II in the regulation of breast cancer cell adhesion and invasion. Endocr Relat Cancer.

[34] Olschewski DN, Hofschröer V, Nielsen N, Seidler DGDG, Schwab A, Stock C (2018). The angiotensin II type 1 receptor antagonist losartan affects NHE1-dependent melanoma cell behavior. Cell Physiol Biochem.

[35] Wu X, Kihara T, Hongo H, Akaike A, Niidome T, Sugimoto H (2010). Angiotensin receptor type 1 antagonists protect against neuronal injury induced by oxygen-glucose depletion. Br J Pharmacol.

[36] Lin Y-C, Boone M, Meuris L, Lemmens I, Van Roy N, Soete A (2014). Genome dynamics of the human embryonic kidney 293 lineage in response to cell biology manipulations. Nat Commun.

[37] Abraham J-A, Linnartz C, Dreissen G, Springer R, Blaschke S, Rueger MA (2018). Directing neuronal outgrowth and network formation of rat cortical neurons by cyclic substrate stretch. Langmuir.

[38] Tanskanen JMA, Kapucu FE, Hyttinen JAK. A line of MEA signal analysis methods for human stem cell-derived and other dynamic neuronal cultures. Front Cell Neurosci. 2018;12.

[39] Habibey R, Latifi S, Mousavi H, Pesce M, Arab-Tehrany E, Blau A (2017). A multielectrode array microchannel platform reveals both transient and slow changes in axonal conduction velocity. Sci Rep.

[40] Hyvärinen T, Hagman S, Ristola M, Sukki L, Veijula K, Kreutzer J (2019). Co-stimulation with IL-1β and TNF-α induces an inflammatory reactive astrocyte phenotype with neurosupportive characteristics in a human pluripotent stem cell model system. Sci Rep.

[41] Habibey R, Golabchi A, Latifi S, Difato F, Blau A (2015). A microchannel device tailored to laser axotomy and long-term microelectrode array electrophysiology of functional regeneration. Lab Chip.

[42] Kakar SS, Sellers JC, Devor DC, Musgrove LC, Neill JD (1992). Angiotensin II type-1 receptor subtype cDNAs: differential tissue expression and hormonal regulation. Biochem Biophys Res Commun.

[43] Chen Y, Morris M (2001). Differentiation of brain angiotensin type 1a and 1b receptor mRNAs a specific effect of dehydration. Hypertension.

[44] Benicky J, Hafko R, Sanchez-Lemus E, Aguilera G, Saavedra JM (2012). Six commercially available angiotensin II AT1 receptor antibodies are non-specific. Cell Mol Neurobiol.

[45] Hafko R, Villapol S, Nostramo R, Symes A, Sabban EL, Inagami T (2013). Commercially available angiotensin II At₂ receptor antibodies are nonspecific. PLoS ONE.

[46] Ma TK, Xu L, Lu LX, Cao X, Li X, Li LL (2019). Ursolic acid treatment alleviates diabetic kidney injury by regulating the ARAP1/AT1R signaling pathway. Diabetes Metab Syndr Obes.

[47] Khan N, Muralidharan A, Smith MT (2017). Attenuation of the infiltration of angiotensin II expressing CD3+ T-cells and the modulation of nerve growth factor in lumbar dorsal root ganglia—a possible mechanism underpinning analgesia produced by EMA300, an angiotensin II Type 2 (AT2) receptor antagonist. Front Mol Neurosci.

[48] Sasamura H, Hein L, Krieger JE, Pratt RE, Kobilka BK, Dzau VJ (1992). Cloning, characterization, and expression of two angiotensin receptor (AT-1) isoforms from the mouse genome. Biochem Biophys Res Commun.

[49] Timmermans PB, Wong PC, Chiu AT, Herblin WF, Benfield P, Carini DJ, et al. Angiotensin II receptors and angiotensin II receptor antagonists. Pharmacol Rev. 1993;45. 8372104

[50] Saavedra JM, Sánchez-Lemus E, Benicky J (2011). Blockade of brain angiotensin II AT1 receptors ameliorates stress, anxiety, brain inflammation and ischemia: therapeutic implications. Psychoneuroendocrinology.

[51] Wang J, Pang T, Hafko R, Benicky J, Sanchez-Lemus E, Saavedra JM (2014). Telmisartan ameliorates glutamate-induced neurotoxicity: roles of AT 1 receptor blockade and PPARγ activation. Neuropharmacology.

[52] Yaginuma H, Kawai S, Tabata KV, Tomiyama K, Kakizuka A, Komatsuzaki T (2014). Diversity in ATP concentrations in a single bacterial cell population revealed by quantitative single-cell imaging. Sci Rep.

[53] Xing LY, Yang T, Cui S Sen, Chen G. Connexin hemichannels in astrocytes: role in CNS disorders. Front Mol Neurosci. 2019;12. 10.3389/fnmol.2019.00023 PMC6372977 30787868

[54] Furness DN, Dehnes Y, Akhtar AQ, Rossi DJ, Hamann M, Grutle NJ (2008). A quantitative assessment of glutamate uptake into hippocampal synaptic terminals and astrocytes: new insights into a neuronal role for excitatory amino acid transporter 2 (EAAT2). Neuroscience.

[55] Rose CR, Ziemens D, Untiet V, Fahlke C (2018). Molecular and cellular physiology of sodium-dependent glutamate transporters. Brain Res Bull.

[56] Sharma A, Kazim SF, Larson CS, Ramakrishnan A, Gray JD, McEwen BS (2019). Divergent roles of astrocytic versus neuronal EAAT2 deficiency on cognition and overlap with aging and Alzheimer’s molecular signatures. Proc Natl Acad Sci.

[57] Kobayashi T, Kawamata T, Shibata N, Okada Y, Kobayashi M, Hori T (2009). Angiotensin II type 1 receptor blocker telmisartan reduces cerebral infarct volume and peri-infarct cytosolic phospholipase A2 level in experimental stroke. J Neurotrauma.

[58] Li T, Chen X, Zhang C, Zhang Y, Yao W (2019). An update on reactive astrocytes in chronic pain. J Neuroinflammation.

[59] Stoll M, Steckelings UM, Paul M, Bottari SP, Metzger R, Unger T (1995). The angiotensin AT2-receptor mediates inhibition of cell proliferation in coronary endothelial cells. J Clin Investig.

[60] Meffert S, Stoll M, Steckelings UM, Bottari SP, Unger T (1996). The angiotensin II AT2 receptor inhibits proliferation and promotes differentiation in PC12W cells. Mol Cell Endocrinol.

[61] Ager EI, Chong WW, Wen S wen, Christophi C. Targeting the angiotensin II type 2 receptor ( AT2R ) in colorectal liver metastases. Cancer Cell Int. 2010;10:1–12. 10.1186/1475-2867-10-19 PMC2902462 20584290

[62] Kettenmann H, Kirchhoff F, Verkhratsky A (2013). Microglia: new roles for the synaptic stripper. Neuron.

[63] Lee H-G, Wheeler MA, Quintana FJ (2022). Function and therapeutic value of astrocytes in neurological diseases. Nat Rev Drug Discov.

[64] Habib N, McCabe C, Medina S, Varshavsky M, Kitsberg D, Dvir-Szternfeld R (2020). Disease-associated astrocytes in Alzheimer’s disease and aging. Nat Neurosci.

[65] Wheeler MA, Clark IC, Tjon EC, Li Z, Zandee SEJ, Couturier CP (2020). MAFG-driven astrocytes promote CNS inflammation. Nature.

[66] Clarke LE, Barres BA (2013). Emerging roles of astrocytes in neural circuit development. Nat Rev Neurosci.

[67] Mohseni Ahooyi T, Shekarabi M, Decoppet EA, Langford D, Khalili K, Gordon J (2018). Network analysis of hippocampal neurons by microelectrode array in the presence of HIV-1 Tat and cocaine. J Cell Physiol.

[68] Mahmoud S, Gharagozloo M, Simard C, Gris D (2019). Astrocytes maintain glutamate homeostasis in the CNS by controlling the balance between glutamate uptake and release. Cells.

[69] Li Q, Marcu DC, Palazzo O, Turner F, King D, Spires-Jones TL (2020). High neural activity accelerates the decline of cognitive plasticity with age in Caenorhabditis elegans. Elife.

[70] Falcucci RM, Wertz R, Green JL, Meucci O, Salvino J, Fontana ACK (2019). Novel positive allosteric modulators of glutamate transport have neuroprotective properties in an in vitro excitotoxic model. ACS Chem Neurosci.

[71] Costa-Besada MA, Valenzuela R, Garrido-Gil P, Villar-Cheda B, Parga JA, Lanciego JL (2018). Paracrine and intracrine angiotensin 1–7/Mas receptor axis in the substantia nigra of rodents, monkeys, and humans. Mol Neurobiol.

