# Supplementary material for: The angiotensin II receptors type 1 and 2 modulate astrocytes and their crosstalk with microglia and neurons in an in vitro model of ischemic stroke
Source: BMC Neurosci. 2024 Jun 26;25:29. doi: 10.1186/s12868-024-00876-x (PMC11202395; doi:10.1186/s12868-024-00876-x)
Supplement: Supplementary file 1 — Additional file 1: Figure S1. [file 12868_2024_876_MOESM1_ESM.docx]

**
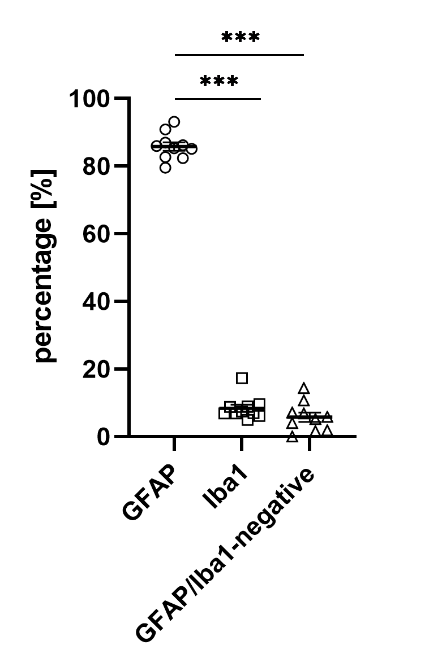
**

**
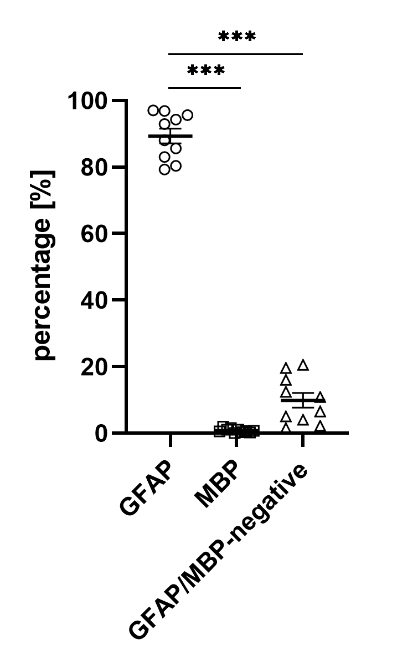
**

* p < 0.05, ** p < 0.01, and *** p < 0.001 compared different experimental groups as marked by horizontal bar; graphs depict mean values ± standard error of the mean (SEM).

A) Astrocyte culture was stained for GFAP (green) and Iba1 (red) in order to rule out any major contamination by microglia. Hoechst stained all cell nuclei blue; scale bars = 100 µm.

B) GFAP-positive cells represented 86% of the total cells, whereas Iba1-positive cells represented 8% (GFAP=86% vs. Iba1=8%, n=10/group; ANOVA, Dunnett’s post hoc test: p<0.001).

C) Astrocyte culture was stained for GFAP (green) and MBP in order to rule out any major contamination by oligodendrocytes. Hoechst stained all cell nuclei blue; scale bars = 100 µm.

D) GFAP-positive cells represented 89% of the total cells, whereas MBP-positive cells represented 0.8% (GFAP=89% vs. MBP=0.8%, n=10/group; ANOVA, Dunnett’s post hoc test: p<0.001).
