# Supplementary material for: The angiotensin II receptors type 1 and 2 modulate astrocytes and their crosstalk with microglia and neurons in an in vitro model of ischemic stroke
Source: BMC Neurosci. 2024 Jun 26;25:29. doi: 10.1186/s12868-024-00876-x (PMC11202395; doi:10.1186/s12868-024-00876-x)
Supplement: Supplementary file 2 — Additional file 2: Figure S2. [file 12868_2024_876_MOESM2_ESM.docx]

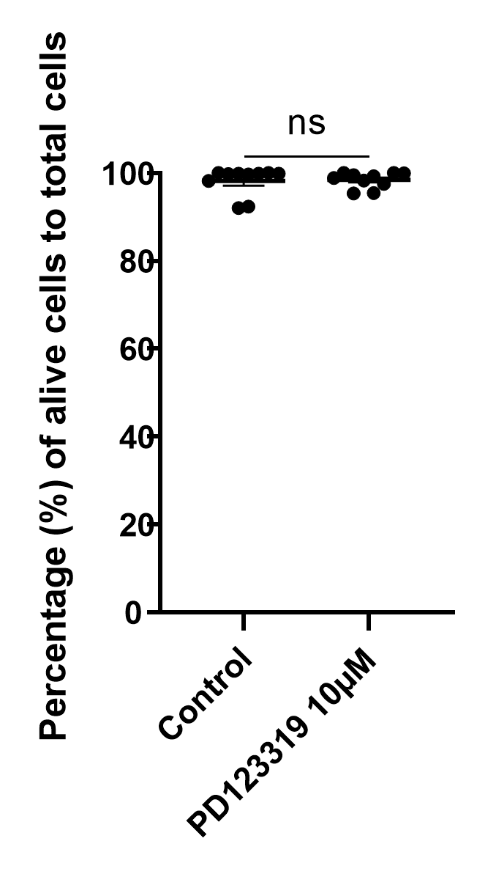
A

* p < 0.05, ** p < 0.01, and *** p < 0.001 compared different experimental groups as marked by horizontal bar; graphs depict mean values ± standard error of the mean (SEM).

A) Incubation with 10 µM PD123319 over 48 hours did not affect percentage of alive to total cells in comparison to untreated control cells (Control=98% vs. PD123319=98%, n=10/group; t-test<0.001).
