# Supplementary material for: The angiotensin II receptors type 1 and 2 modulate astrocytes and their crosstalk with microglia and neurons in an in vitro model of ischemic stroke
Source: BMC Neurosci. 2024 Jun 26;25:29. doi: 10.1186/s12868-024-00876-x (PMC11202395; doi:10.1186/s12868-024-00876-x)
Supplement: Supplementary file 3 — Additional file 3: Figure S3. [file 12868_2024_876_MOESM3_ESM.docx]

* p < 0.05, ** p < 0.01, and *** p < 0.001 compared different experimental groups as marked by horizontal bar; graphs depict mean values ± standard error of the mean (SEM).

A) Intensity parameters of immunofluorescent staining of S100A10 in GFAP positive astrocytes were analyzed using a custom MATLAB (Mathworks, 2021b) script using a mask of GFAP positive pixels using Otsu’s method. A trend of increase of S100A10 intensity was observed for astrocytes exposed to 2 hours of OGD (Control=31.5 vs. Control+OGD=62.8; ANOVA, followed by Dunnett’s post hoc test: n.s.; Control=31.5 vs. PD123319=37.1; ANOVA, followed by Dunnett’s post hoc test: n.s.).

B) Intensity parameters of immunofluorescent staining of iNOS in Iba1 positive microglia was analzyed as mentiond in supplemental figure 3A. A significant increase for iNOS for microglia treated with CM PD123319 was observed compared to CM Control (CM Control=37.4 vs. CM PD123319=68; ANOVA, followed by Dunnett’s post hoc test: p<0.01; CM Control=37.4 vs. Control=33.1; ANOVA, followed by Dunnett’s post hoc test: n.s.; CM Control=37.4 vs. CM Telmisartan=17.1; ANOVA, followed by Dunnett’s post hoc test; n.s.).
