# Supplementary material for: The angiotensin II receptors type 1 and 2 modulate astrocytes and their crosstalk with microglia and neurons in an in vitro model of ischemic stroke
Source: BMC Neurosci. 2024 Jun 26;25:29. doi: 10.1186/s12868-024-00876-x (PMC11202395; doi:10.1186/s12868-024-00876-x)
Supplement: Supplementary file 4 — Additional file 4: Figure S4. [file 12868_2024_876_MOESM4_ESM.docx]

Representative raster plots (left) and voltage traces (right) of cortical neurons on MEAs exposed to CM Control (A), CM Telmisartan (B) and CM PD123319 (C) at the 24-hour time point after exposure.
